# Supplementary figures and images for: Evaluation of dental pulp stem cells response to flowable nano-hybrid dental composites: A comparative analysis
Source: PLoS One. 2024 May 13;19(5):e0303154. doi: 10.1371/journal.pone.0303154 (PMC11090312; doi:10.1371/journal.pone.0303154)

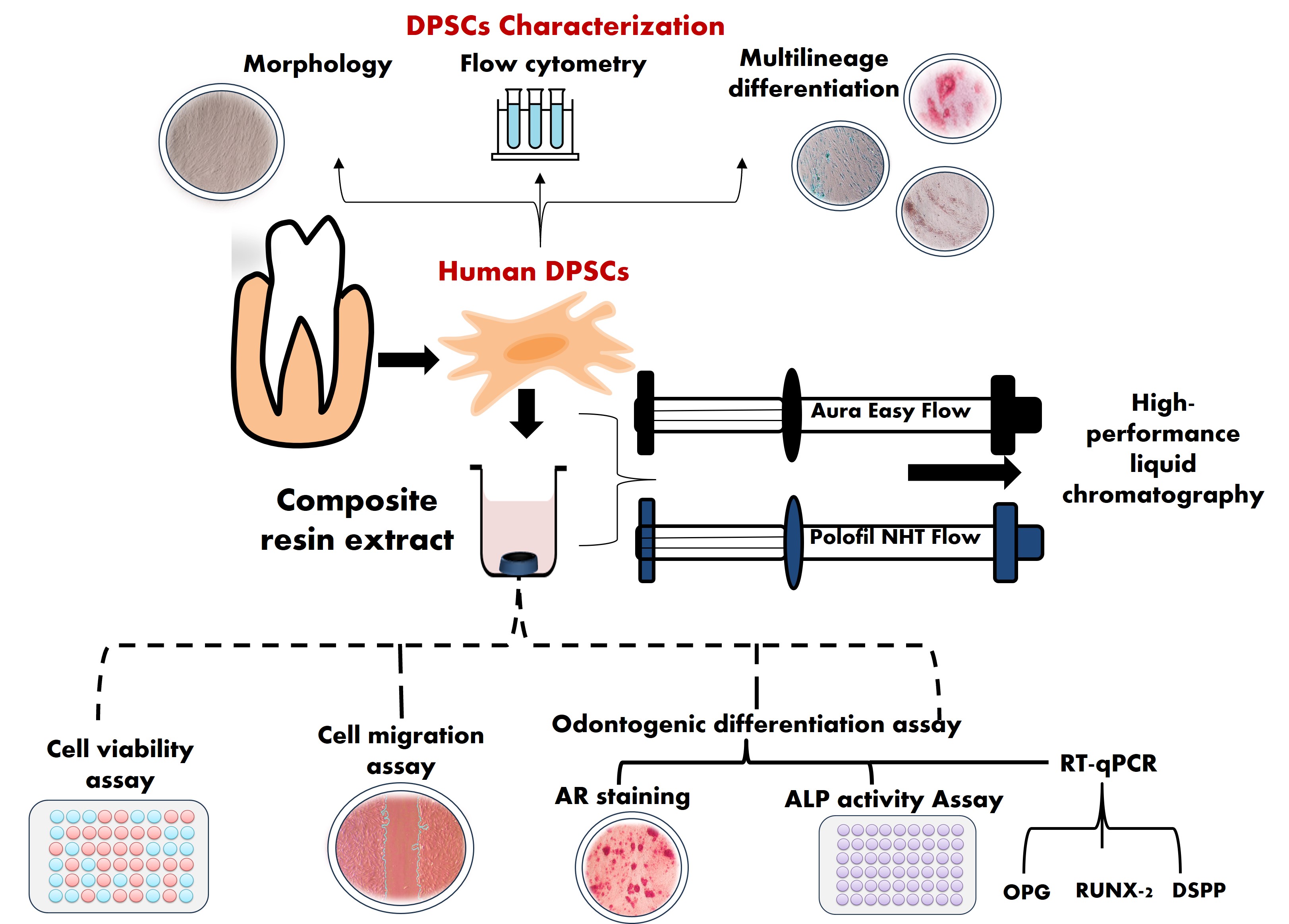

Supplement: S1 Graphical abstract — (JPG) [file pone.0303154.s007.jpg]
