# Supplementary material for: Evaluation of dental pulp stem cells response to flowable nano-hybrid dental composites: A comparative analysis
Source: PLoS One. 2024 May 13;19(5):e0303154. doi: 10.1371/journal.pone.0303154 (PMC11090312; doi:10.1371/journal.pone.0303154)
Supplement: Attachment — Submitted filename: 24.12 PLOS ONE.docx [file pone.0303154.s008.docx]

Thank you for the authors for addressing this important point of research and for their efforts. However, I don’t recommend publishing of this paper in the current version because of the following causes:

1) The quality of figures is too pore; the typing is not clear and the colors of the figures is not appropriate
2) The microscopic images have poor quality with no scale bars
3) The overall English and scientific writing of the paper is not professional and should be totally revised
4) Many sections in the methodology are not mentioned clearly.
