# Supplementary material for: Evaluation of dental pulp stem cells response to flowable nano-hybrid dental composites: A comparative analysis
Source: PLoS One. 2024 May 13;19(5):e0303154. doi: 10.1371/journal.pone.0303154 (PMC11090312; doi:10.1371/journal.pone.0303154)
Supplement: Attachment — Submitted filename: Response to reviewers.docx [file pone.0303154.s009.docx]

Response to reviewers

The authors would like to acknowledge the insightful feedback and suggestions regarding our manuscript. We have carefully considered each of your points and have made the required revisions (marked in the manuscript).

Reviewer #1:

A. A scheme depicting the whole picture of this study could be added at the end of Introduction.

Response: A graphical abstract is added as figure 10.

B. For the chemicals listed in Table 2, it would be helpful to also provide the molecular structures.

Response: The molecular structure is added to Table 2.

C. Scale bars should be supplemented in Figure 1A, Figure 2 and Figure 7.

Response: scale bars added.

D. What was the unit of data in Table 4? Please demonstrate in the columns.

Response: the unit of data is µg/ml, added to Table 4 and Figure 4.

E. Statistical analysis should be performed for Figure 4.

Response: statistical analysis of table 4 is presented in table 4.

F. Please include some discussion about clinical and industrial translation in Discussion Section.

Response: added.

G. The Conclusion Section was a bit short. Some critical data could be added therein.

Response: conclusion re-written.

Reviewer #2:

1) The quality of figures is too poor; the typing is not clear and the colors of the figures is not appropriate

Response: replaced with new figures with better resolution.

2) The microscopic images have poor quality with no scale bars

Response: images replaced and scale bars added.

3) The overall English and scientific writing of the paper is not professional and should be totally revised

Response: We have undertaken a comprehensive revision of the manuscript. This revision was conducted with the assistance of a professional scientific editor to ensure that the language is not only grammatically correct but also conveys our research effectively and professionally.

4) Many sections in the methodology are not mentioned clearly

Response: The methodology has been updated to include more description to ensure that the methodology is transparent and reproducible.
