# Supplementary material for: Evaluation of dental pulp stem cells response to flowable nano-hybrid dental composites: A comparative analysis
Source: PLoS One. 2024 May 13;19(5):e0303154. doi: 10.1371/journal.pone.0303154 (PMC11090312; doi:10.1371/journal.pone.0303154)
Supplement: Attachment — Submitted filename: Response to reviewers.docx [file pone.0303154.s010.docx]

We would like to express our gratitude for the time and effort you have dedicated to reviewing our manuscript. We appreciate the insightful comments and constructive feedback provided, which have been instrumental in enhancing the quality and clarity of our work. In response to the comments, we have carefully revised our manuscript, addressing each point to ensure that our study meets the high standards of PLOS ONE.

1. **The abstract should be divided into background, methodology, results and conclusion. Please adjust the subheadings accordingly**: "Thank you for your suggestion. The abstract has been revised to include subheadings for Background, Methodology, Results, and Conclusion, as requested."
2. **Please state in the abstract which odontogenic markers did you evaluate?**: "We appreciate your attention to detail. In the revised abstract, we have clearly stated the odontogenic markers evaluated in our study.
3. **Please provide a list of abbreviations**: "A list of abbreviations used throughout the manuscript has been created and added as a supplementary file."
4. **Please clarify what is % wt when being used for the first time?**: "We have clarified the term '% wt' at its first use in the text to indicate 'percentage by weight'.
5. **The file that I received contains two versions of the manuscript, please edit.** " Following PLOS ONE guidelines, we have included both a marked-up version showing the changes made and an unmarked version in the revised submission."
6. **The overall English of the paper looks better but it still needed to be improved, please consider academic English revision.** "We have taken your advice and have had the manuscript reviewed and revised by a native English-speaking academic in our field to improve the academic English quality."
7. **Line 8 under the subtitle of “DPSCs isolation and culture” in the methodology, there is nothing called 2mm3, please edit.**: "The phrase has been adjusted."
8. **At the end of the same section, there is nothing called “the experiments used cells….”**: "The confusing statement at the end of the 'DPSCs isolation and culture' section has been removed and replaced with a clear description of the cell preparation for experiments."
9. **In the methodology, under the subtitle of “DPSCs Characterization’, please provide the model of the used inverted microscope.** "The model of the inverted microscope used for DPSCs characterization is now specified".
10. **In the flow cytometry part in the methodology, please describe whether the used antibodies were conjugated or not and use the professional way of writing as cells were stained with……..**: "We have revised the section on flow cytometry to specify that the antibodies used were fluorescein-conjugated and have adopted the suggested professional phrasing for cell staining."
11. **In the multilineage differentiation, please add the word of “according to manufacturer’s instructions after the multilineage kit, then briefly describe the performed procedures.**: "Following your suggestion, we have added 'according to the manufacturer’s instructions' after mentioning the multilineage differentiation kit and provided a brief description of the procedures performed."
12. **The methodology still contains many English errors. For example, It is culture medium not culture media**: "We have carefully reviewed the methodology section to correct the English language errors, including the correct use of 'culture medium'."
13. **Under the methodology of the cell viability assay, what are the different time points at which did you added the MTT?**: "The different time points at which the MTT assay was conducted are now clearly stated in the methodology section."
14. **Please follow the professional English terminologies and formatting while writing the methodology, for example; the sub heading under a specific heading should be shifted to some extend and so on.**: " Thank you for your feedback regarding the formatting of the methodology section. We would like to clarify that the formatting of the entire manuscript, including the methodology section, was meticulously done according to the specific instructions and template provided by the journal. We ensured that all headings, subheadings, and text alignments were in strict adherence to these guidelines to maintain consistency and comply with the journal's requirements."
15. **Please provide the name and origins of the companies for the used material and supplies.** "The names and origins of the companies for all used materials and supplies are now included in the methodology section."
16. **The resolution of figure 1 is not clear, A and B have different back grounds and even the histograms are not well- aligned and don’t have the same size.**: "We have enhanced the resolution of Figure 1 and ensured uniform backgrounds and alignment of histograms for clarity."
17. **Please provide a professional presentation for Fig.2** "Figure 2 has been professionally redesigned for clarity and improved presentation."
18. **The resolution and background of fig.5 is not clear.**: "The resolution and background issues in Figure 5 have been addressed to ensure clear visualization."
19. **Figure Legends**: " According to the journal's submission instructions: Figure captions and legends must be inserted in the text of the manuscript, immediately following the paragraph in which the figure is first cited, figure legends are embedded directly within the manuscript at the locations of the corresponding figures, rather than being listed at the end or in a separate section."
